# Investigating genetic overlap of multidimensional pain and suicidal behaviors in >2 million individuals

**DOI:** 10.21203/rs.3.rs-9217629/v1

**Published:** 2026-06-02

**Authors:** Seonggyun Han, Sylvanus Toikumo, Sarah Colbert, Niamh Mullins, Alexander S. Hatoum, Emily DiBlasi, Robert Krueger, Rachel Kember, Andrey Shabalin, Henry Kranzler, Anna Docherty

**Affiliations:** University of Utah School of Medicine; University of Pennsylvania; Icahn School of Medicine at Mount Sinai; Icahn School of Medicine; Washington University Physicians; University of Utah School of Medicine; University of Minnesota; University of Pennsylvania School of Medicine; University of Utah School of Medicine; University of Pennsylvania Perelman School of Medicine

## Abstract

Although chronic pain is a major risk factor for suicidal behaviors, the shared genetic underpinnings of these phenotypes are poorly understood. This study leveraged genome-wide association data to examine the shared genetic architecture between multidimensional pain (MP), capturing broad genetic liability to chronic pain (*n* = 1,235,695), and suicidal behaviors, including suicide attempt (SA, *n* = 787,974) and suicide death (SD, *n* = 18,223). Integrative cross-trait genetic analyses revealed substantial polygenic overlap between MP and both suicidal behaviors, and identified 76 (244 genes) and 10 (27 genes) distinct shared loci for SA and SD, respectively. The genes mapped to the shared SA loci were enriched in neuronal and synaptic pathways, and integration of multi-omics data further prioritized 10 genes, providing convergent molecular context relevant to the neurobiological and behavioral pathways implicated in the association between pain liability and SA. Mendelian randomization analyses supported a potential causal relationship between MP and both suicidal behaviors, with asymmetric bidirectional effects for MP and SA. Mediation analyses further indicated that 14 health-related traits, including psychological and substance-related behaviors, partially mediated the association between MP and SA. Together, these findings reveal shared genetic architecture between MP and suicidal behaviors, with the association partially mediated by convergent neurobiological and potentially modifiable behavioral pathways relevant to prevention and therapeutic efforts.

## Introduction

Suicide is a major public health crisis contributing to millions of deaths worldwide. It is a complex behavior with significant heritability, and a clinical outcome influenced by a broad range of psychiatric and medical conditions. Although research on psychiatric conditions has advanced our understanding of suicide risk mechanisms, the broader landscape of suicide risk factors in the context of medical conditions has not yet been fully characterized. Notably, recent studies have underscored the role of physical health problems, which represent the most common class of comorbidities among suicide decedents^1^.

Among physical health conditions, chronic pain has emerged as a significant risk factor for suicide^2,3^. It is uniquely associated with risk for suicide even after controlling for co-occurring psychiatric conditions^4^, with suicide risk increasing two-fold in individuals with chronic pain^5^. Previous clinical and epidemiological studies have established that risk of suicide is elevated in individuals with various pain conditions, such as multisite musculoskeletal pain, knee pain, back pain, psychogenic pain, and fibromyalgia^6-10^. Despite this well-established association, strategies to reduce suicide risk attributable to chronic pain remain limited, largely due to an incomplete understanding of mechanisms linking chronic pain and suicidal behaviors, which in turn hampers the identification of individuals with chronic pain who are at particularly high risk of suicide.

There is emerging evidence that biological mechanisms underlying chronic pain may contribute to suicide risk, and that accounting for genetic variation underlying pain may help to reduce the genetic heterogeneity of suicide. Multiple types of chronic pain are strongly linked to nervous system and brain-related processes, including synaptic plasticity, brain structure and function, precuneus thickness, and glutamatergic activity, which have also been implicated in suicide risk^9,11-14^. Importantly, a population-based Swedish twin study showed that the association between chronic pain and suicide may be driven largely by underlying shared genetic factors, rather than solely by the phenotypic effects of chronic pain on suicide risk^15^. In that study, chronic pain increased the risk of suicide in unrelated individuals, while the association was attenuated in dizygotic twin pairs and disappeared in monozygotic twin pairs, suggesting a substantial contribution of shared genetic liability^15^. Consistent with this, cross-disease polygenic score (PGS) analyses identified significant genetic covariation between chronic pain and suicide death, independent of chronic pain diagnostic history, supporting a shared genetic basis for these traits^16^. However, genetic studies of chronic pain and suicide risk are limited and primarily capture genome-wide polygenic overlap.

A major challenge in characterizing the role of chronic pain in suicide risk lies in the complexity and heterogeneity of chronic pain phenotypes themselves. There are multiple dimensions of chronic pain, including intensity, duration, anatomical site, and measurement approaches (self-reported symptoms, clinical diagnoses, and ICD codes), which vary substantially across studies^15,17^. In addition, multiple chronic pain conditions frequently co-occur within individuals^15,18-20^. These factors likely contribute to inconsistent findings across studies, hindering our understanding of whether suicide risk is linked to specific pain subtypes or to a shared, overarching liability to chronic pain. Thus, it is important to also investigate a broad, general genetic factor underlying chronic pain to elucidate the biological pathways that link chronic pain and suicidal behavior. However, the few genetic studies examining the association between chronic pain and suicide risk to date have been restricted to specific individual pain phenotypes^16,17^. Thus, the full scope of the shared genetic architecture and underlying biology linking chronic pain and suicide remains incompletely characterized.

In addition, chronic pain is associated with a range of health-related phenotypes, including psychological distress, psychiatric conditions, and other adverse behavioral outcomes, many of which have also been implicated in suicidal behaviors^21-23^. However, whether such traits mediate the relationship between genetic liability for chronic pain and suicide risk remains largely unexplored.

**Figure 1:**
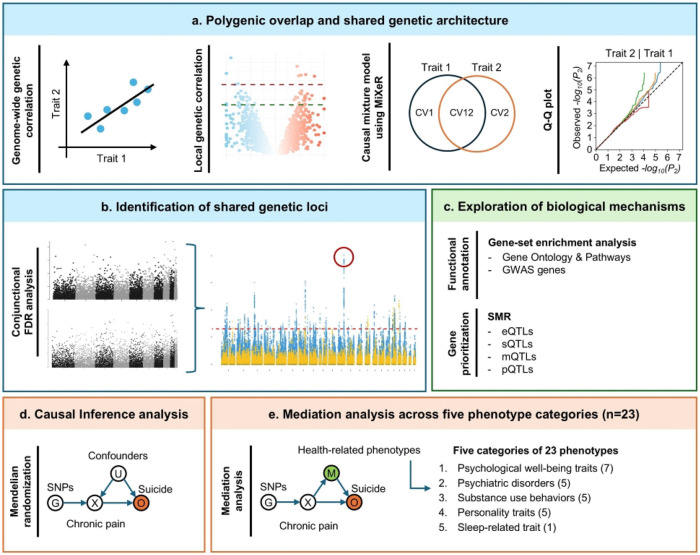
Overall study design and analytic framework. **(a)** Polygenic overlap and shared genetic architecture. Genome-wide and local polygenic overlap between chronic pain and suicidal behaviors (examining suicide attempt and suicide death separately) was evaluated using LDSC-based global genetic correlation, LAVA, and MiXeR, and conditional Q-Q plot. **(b)** Identification of shared genetic loci. Conjunctional false discovery rate (conjFDR) analysis was applied to jointly leverage GWAS signals for MP and suicide attempt/death, identifying specific shared genetic loci beyond conventional genome-wide significance thresholds. **(c)** Identification of putative molecular pathways underlying shared loci. Genes mapped to shared loci were investigated to evaluate the molecular and biological processes underlying the shared genetic architecture by performing gene-set and pathway enrichment analyses. Multi-omics integration using SMR was conducted to prioritize credible genes and regulatory mechanisms. **(d)** Causal inference analyses. Mendelian randomization analyses were conducted to assess putative causal relationships between chronic pain and suicide attempt/death. **(e)** Mediation analyses. Mediation analyses were subsequently performed to evaluate the potential mediating roles of 23 health-related phenotypes that are plausibly associated with both multidimensional pain and suicide, including five previously identified conceptual categories: 1) psychological well-being traits (irritable mood, feeling lonely, feeling miserable, feeling nervous, feeling tense, mental well-being, and prolonged worry), 2) psychiatric disorders (attention-deficit/hyperactivity disorder, bipolar disorder, major depressive disorders, post-traumatic stress disorder, and schizophrenia), 3) substance-related behaviors (alcohol use disorder, cannabis use disorder, opioid medication, problematic alcohol use, and smoking), 4) personality traits (agreeableness, conscientiousness, extraversion, neuroticism, and openness), and 5) sleep-related trait (insomnia).

To address these critical knowledge gaps, we conducted a comprehensive cross-trait genomic analysis of multidimensional pain (MP) and suicidal behavior (including suicide attempt (SA) and suicide death (SD)) using the large-scale available genome-wide association studies (GWAS). The MP GWAS (n = 1,235,695) represents a broad, general genetic liability to multidimensional pain that combines multiple pain dimensions including pain intensity, chronicity, spatial distribution, and diagnosis-based pain codes across diverse pain assessment frameworks^24^. The GWASs of SA^25^ and SD^26^ included 787,974 and 18,223, individuals respectively. We applied a suite of statistical and functional genomic analytic tools, including LAVA^27^, MiXeR^28^, conjFDR^29^, SMR^30^, and Mendelian randomization^31^ to delineate the scope of shared genetic architecture, characterize pleiotropy, and detect causal relationships. Here, we characterize the genetic overlap of MP with SA and SD, enhancing our understanding of disease biology and informing future prevention and therapeutic strategies. An analytic overview of this study is presented in Fig. 1.

## Results

### Genetic overlap of multidimensional pain and suicide

We used univariate MiXeR^28^ to estimate polygenicity of each trait. MiXeR estimated 14,911 trait-influencing variants for MP (standard error [SE] = 420), which explain 90% of its SNP heritability (h^2^_SNP_). The corresponding estimates for SA and SD were 8,981 (SE = 850) and 5,436 (SE = 6,454) variants, respectively. The observed-scale SNP heritability estimates inferred by MiXeR were 0.065 (SE = 0.001), 0.014 (SE = 2.0e-4), and 0.129 (SE = 0.012) for MP, SA and SD, respectively. Univariate MiXeR models for MP and SA showed positive differences in Akaike information criterion (AIC) values, indicating better fit of the polygenic mixture model over the infinitesimal model^32^. The SD model yielded a marginally negative AIC difference (−1.18), consistent with limited statistical power, likely due to the smaller sample size. Nevertheless, the heritability estimate was statistically stable (z-score = 10.75), supporting the presence of polygenic signal. We therefore proceeded with exploratory bivariate analyses for SD with cautious interpretation.

**Figure 2:**
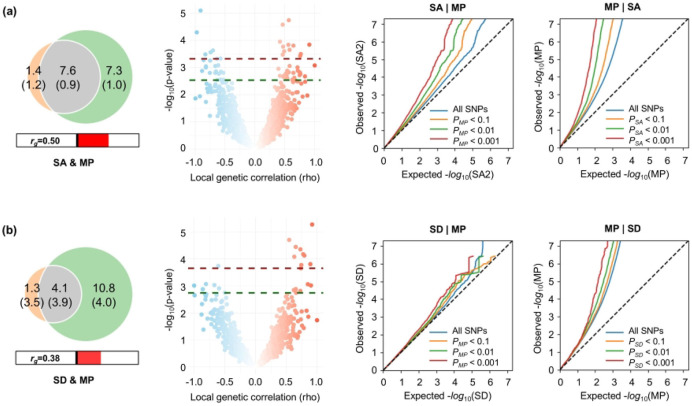
Genome-wide and local-based genetic overlap between multidimensional pain (MP) and **(a)** suicide attempt (SA) and **(b)** suicide death (SD). The Venn diagrams illustrate the estimated number of causal shared variants with standard errors (in thousands) explaining 90% of SNP-based heritability and with the genetic correlation (*r_g_*) from bivariate MiXeR analysis. The middle volcano plots show LAVA local genetic correlation coefficients (rho) on the X-axis and −log_10_ p-values per investigated locus. Red and green dotted horizontal lines indicate FDR<0.05 and<0.1, respectively. The conditional Q-Q plots show nominal versus empirical −log10 p-values of suicide attempt/death at the level of p<1, p<0.1, p<0.01, p<0.001 of multidimensional pain (left) and vice versa (right).

Across methods, MP and suicidal behaviors demonstrated substantial genetic overlap contributing to their co-occurrence, with evidence of some nuances across specific loci. Bivariate MiXeR revealed extensive polygenic overlap between MP and SA, which exceeded that shown by their genetic correlations (*r_g_*) (Fig. 2a). Both AIC differences (i.e., AIC_min_, which compares the best-fitting model to minimum possible overlap and AIC_max_, which compares it to maximum possible overlap) were positive, supporting adequate model fit for estimating genetic overlap. Of the estimated 9.0 k trait-influencing variants for SA, 7.6 k (84.4% with SE = 0.9 k) were shared with MP, corresponding to a Dice coefficient-the fraction of shared variants from the total number of variants-of 64%. Despite the substantial overlap, genome-wide *r_g_*-which reflects the net balance of concordant and discordant effects across the genome-was moderate (*r_g_*=0.50; SE = 0.007). This pattern provides evidence of mixed effect directions contributing to their relationship. This interpretation is supported by the fact that 78.9% of shared trait-influencing variants were concordant in effect direction and both positive and negative local genetic correlations were observed across LD-based regions in Local Analysis of [co]Variant Association (LAVA) (Methods) (see the second middle plot in Fig. 2a). A total of 19 regions were identified as significant in LAVA at FDR < 0.05 (Supplementary Table 1), with FDR < 0.1 results considered suggestive. Q-Q plots conditioning SA on MP and vice versa further demonstrated pleiotropic enrichment, with progressively stronger leftward deflection observed across increasing levels of SNP significance in both directions, indicating substantial polygenic overlap between the two traits (the right plots Fig. 2a).

For SD, MiXeR estimated 4.1 k of 5.4 k trait-influencing variants (75.9% with SE = 3.9 k) were shared with MP, corresponding to a Dice coefficient of 36% (Fig. 2b). However, the MiXeR analysis of this trait showed suboptimal model fit, with negative AIC difference values for both minimum and maximum overlap models and large SE. This indicates that the estimated polygenic overlap is sensitive to model specification^32^, and therefore should be interpreted with caution. Nevertheless, the genome-wide *r_g_* was 0.38 and the proportion of concordant effects was 91.3% within the shared variants, consistent with the majority of significant local genetic correlations being positive (see the second middle plot in Fig. 2b). In total, 11 regions were identified as significant in LAVA at FDR < 0.05 (Supplementary Table 2). The Q-Q plot exhibited polygenic enrichment for MP and SD, but not for SD and MP.

### Shared genetic loci between multidimensional pain and suicide attempt or suicide death

**Figure 3:**
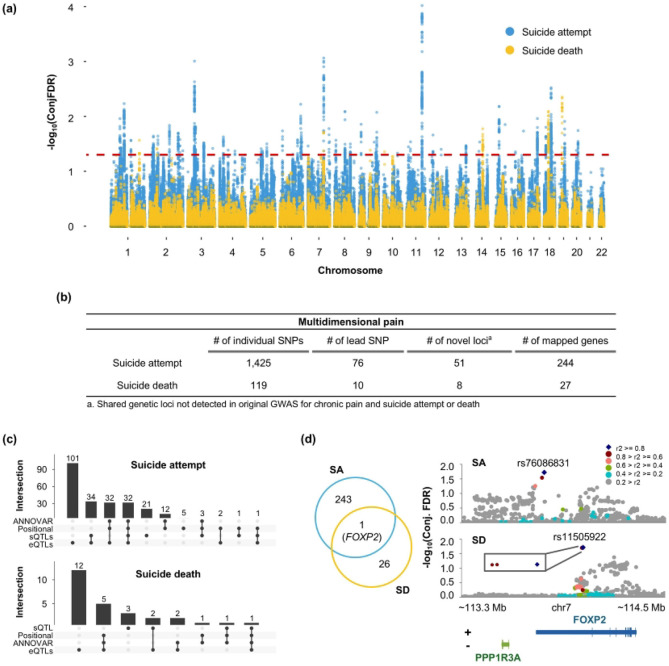
Shared genetic loci between multidimensional pain and suicide attempt or suicide death from conjunctional FDR (conjFDR) analysis. **(a)** Manhattan plots illustrating the −log_10_ transformed conjFDR values for each SNP. A red dotted line indicates conjFDR<0.05. **(b)** The number of significant shared loci, their independent lead SNPs within each linkage disequilibrium (LD) block based on r^2^>0.2, novel SNPs, and mapped genes. Genes were mapped from candidate shared SNPs using 1) ANNOVAR annotation, 2) FUMA positional mapping, 3) FUMA GTEx eQTL mapping, and 4) GTEx sQTL mapping. **(c)** An UpSet plot of intersection among genes in four resources. **(d)** The overlap of genes between suicide attempt and death, and regional plots of conjFDR association statistics at the *FOXP2* locus. The box in the plot for suicide death represents a zoomed-in view of the highlighted locus, showing three SNPs in high LD.

To identify specific shared genetic loci underlying the observed polygenic overlap, we applied conjunctional false discovery rate (conjFDR)^29^ analysis. At a threshold of < 0.05, we identified 76 independent genetic loci (lead SNPs), corresponding to 1,425 individual SNPs, for SA and 10 loci (119 individual SNPs) for SD that were jointly associated with MP (Fig. 3 and Supplementary Tables 3 and 4). The full list of significantly shared SNPs is provided in Supplementary Tables 5 and 6 for SA and SD, respectively. Among the 76 shared loci for SA, 51 did not reach genome-wide significance (p < 5e-8) in the original GWAS of either MP or SA. Of the 10 shared loci for SD, 8 were not significant in the original GWAS of either MP or SD. The most significant genomic risk locus among conjFDR-identified loci for SA was chr11:113218050–113431960 including two LD-independent lead SNPs rs2514218 (conjFDR = 8.80e-5) and rs12798900 (conjFDR = 1.09e-4) (Supplementary Fig. 1a). This locus did not overlap with LAVA-significant regions at FDR < 0.05, but overlapped with regions reaching FDR < 0.1. The most significant locus for SD was chr19:19429220–19480573, including rs10421505 (conjFDR = 4.48e-3) (Supplementary Fig. 1b). This locus did not overlap with any LAVA-significant regions. A total of 7 and 1 conjFDR-identified independent loci for SA and SD, respectively, overlapped with LAVA-significant (FDR < 0.05) regions, supporting convergent evidence of shared genetic effects despite methodological differences between regional correlation and SNP-level enrichment. This overlap suggests that, within these loci, shared SNP-level associations may align with regionally consistent cross-trait effects.

A total of 244 and 27 genes were mapped to candidate loci shared between MP and SA (Fig. 3c and Supplementary Table 7) or SD (Fig. 3c and Supplementary Table 8), respectively, based on positional mapping, ANNOVAR^33^, expression quantitative trait loci (eQTL), and splicing quantitative loci (sQTLs). Only one gene, *FOXP2*, overlapped both the MP-SA and MP-SD analyses, with lead SNPs, rs76086831 and rs11505922, respectively (Fig. 3d). These two SNPs were located within the same genomic risk locus as defined by FUMA^34^, though they were not in linkage disequilibrium (LD, r^2^ = 0.05 in the European population).

### SNP-based and gene-based functional annotation and enrichment analyses

**Figure 4:**
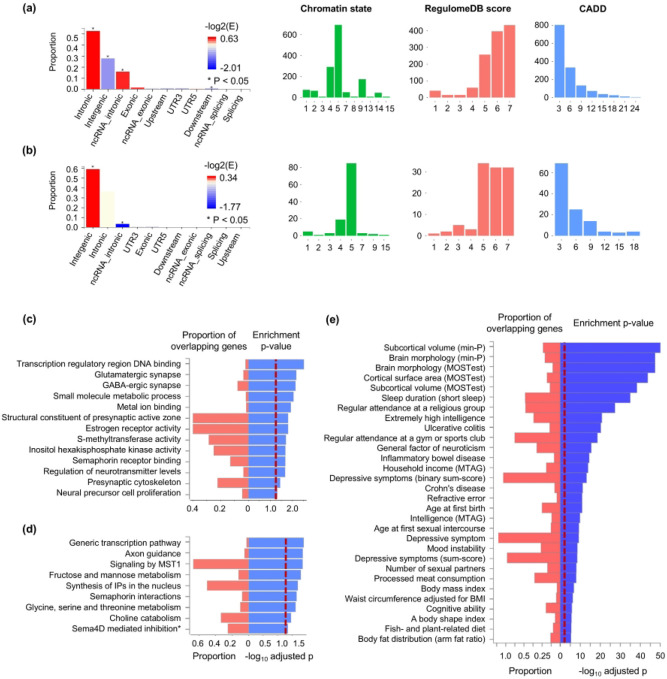
Functional annotation analyses at SNP- and gene-levels with shared loci between multidimensional pain and suicidal behaviors. **(a, b)** Distribution of a functional consequence of candidate shared SNPs for annotations including functional annotation enrichment, RegulomeDB score, chromatin state, and CADD from FUMA for **(a)**suicide attempt and **(b)** suicide death. Gene-set over-representative results for suicide attempt based on **(c)** gene ontology and **(d)** canonical pathways, such as Reactome and KEGG. Sema4D mediated inhibition*= Sema4D mediated inhibition of cell attachment and migration **(e)** GWAS gene set enrichment analysis results (top 30 significant traits).

Functional annotation of shared loci at the SNP-level revealed comparable patterns for SA and SD. For SA, shared loci were over-represented in intronic (56.1%) or non-coding RNA intronic regions (15.9%). A total of 83% were located in open chromatin regions (minimum chromatin states ≤ 7), 5% showed likely regulatory functionality in transcription factor motifs (RegulomeDB scores ≤ 2), and 5% were putatively deleterious (Combined Annotation Dependent Depletion (CADD) score > 12.37) (Fig. 4a). Among the 76 lead SNPs for SA, five (rs17782474, rs72961179, rs76086831, rs6968125, and rs4702) had CADD scores exceeding 12.37, mapped to *AFF3* (intronic), ENSG00000299281 (non-coding RNA annotated as Inc-KLF7-2), *FOXP2* (intronic), ENSG00000298119 (non-coding RNA annotated as LINC01392), and *FURIN* (3’ UTR), respectively. Several of these loci were not genome-wide significant in the original GWAS, suggesting novel cross-trait associations.

For SD, shared loci were primarily located in intergenic (58.8%). Consistent with SA, 97% were situated in open chromatin regions, 3% were located in transcription factor motifs, and 5% were putatively deleterious (CADD > 12.37) (Fig. 4b). One (rs12433032) of the 10 lead SNPs exceeded the CADD threshold and mapped to *GALNT16* (intronic), a locus previously identified in the MP GWAS but not in the original SD GWAS, highlighting its cross-trait implication.

To gain insight into the biological and molecular roles of the 244 genes mapped from shared loci for SA, we performed gene-set enrichment analyses using ConsensusPathDB (CPDB)^35^. These analyses were restricted to SA given the limited number of genes identified for SD. Gene ontology (GO) enrichment analysis revealed 32 GO terms related to neural functions, including neural precursor cell proliferation, synapse organization, semaphorin receptor binding, regulation of neurotransmitter levels, GABAergic synapse, glutamatergic synapse, and presynaptic cytoskeleton (Fig. 4c and Supplementary Table 9). The pathway over-representative analysis identified 14 enriched pathways, including signaling by MST1, axon guidance, semaphorin interactions, glycine, serine and threonine metabolism, and choline catabolism (Fig. 4d and Supplementary Table 10). In addition, GWAS signals of brain structure- and function-related phenotypes (e.g., subcortical volume and brain morphology), sleep duration, neuroticism, depressive symptoms, and mood instability were significantly enriched among genes shared between MP and SA (Fig. 4e and Supplementary Table 11).

### Gene prioritization

**Figure 5:**
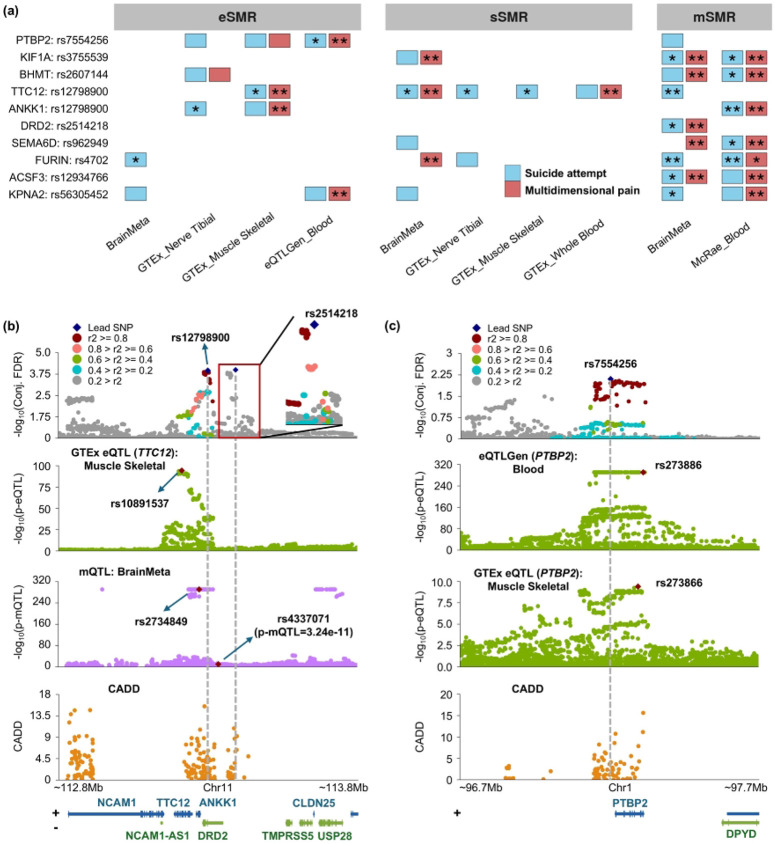
Gene prioritization of suicide attempt-multidimensional pain shared genetic loci via summary-data-based Mendelian randomization (SMR) analysis with eQTL, sQTL, and mQTL resources. **(a)** The x-axis shows QTL databases and the y-axis indicates gene and lead SNP. Blue and red refer to suicide attempt and multidimensional pain, respectively. Symbols indicate the level of statistical significance: * FDR<0.1; ** FDR<0.05; colored boxes without symbols indicate nominal p-value<0.05. **(b** and **c)** Regional plots exhibiting conjFDR, p-values of QTLs: green, purple, orange colors refer eQTL, mQTL, and CADD, respectively. In panel b, rs10891537 and rs2734849 are in the same LD block as rs12798900, while rs4337071 is in the LD block tagged by rs2514218. The −log_10_ p-values were capped at 300 for visualization purposes; values exceeding 300 were plotted as 300.

To further prioritize putative effector genes, we conducted summary data-based Mendelian randomization (SMR)^30^ analyses integrating GWAS signals for MP and SA with eQTL, sQTL, methylation QTL (mQTL), and protein QTL (pQTL) datasets. These analyses identified 9 distinct genetic loci, corresponding to 10 candidate genes supported by at least one of the four QTL categories across both MP and SA (Fig. 5a and Supplementary Table 12); there was no significant SMR result in pQTL. Notably, these included the top conjFDR locus on chromosome 11, where convergent SMR evidence prioritized *TTC12*, *ANKK1*, and *DRD2* within two LD blocks tagged by rs12798900 and rs2514218 (Fig. 5b). *PTBP2* was also supported in the conjFDR locus harboring rs7554256, which represented the second most significant shared region among the SMR-prioritized loci (Fig. 5c). For SD, there were no significant SMR genes among 27 genes mapped to the shared loci with MP.

### Genetically Informed Causal Relationships via Mendelian Randomization

**Figure 6:**
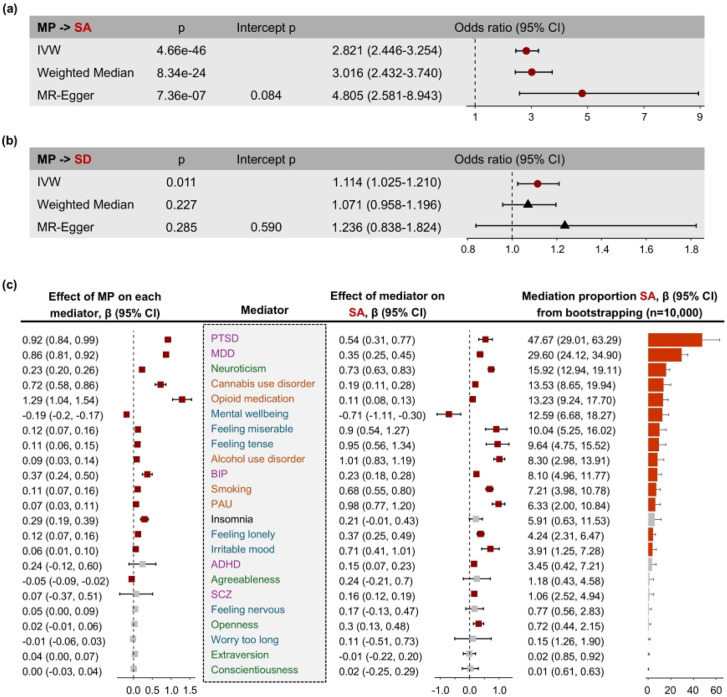
Mendelian randomization (MR) analysis. **(a)** MR result from multidimensional pain (MP) to suicide attempt (SA). **(b)** MR result from multidimensional pain to suicide death (SD). **(c)** Potential mediating roles of 23 health-related phenotypes plausibly associated with both multidimensional pain and suicidal behavior, including five previously defined conceptual categories: 1) psychological well-being traits (blue colored letters); 2) psychiatric disorders (purple); 3) substance-related behaviors (orange); 4) personality traits (green); and 5) sleep-related trait (black). ADHD: attention-deficit/hyperactivity disorder; BIP: bipolar disorder; MDD: major depressive disorders; PAU: problematic alcohol use; PTSD: post-traumatic stress disorder; SCZ: schizophrenia.

We found evidence of putative causal relationships from MP to both SA and to SD using Mendelian randomization (MR) analyses (Fig. 6). For MP effect on SA, the inverse variance weighted (IVW) method yielded an odds ratio (OR) of 2.82 (95% confidence interval [CI], 2.45–3.25; p = 4.66e-46) (Fig. 6a). Complementary MR methods, including the weighted median (WM) and MR-Egger approaches, provided consistent estimates, supporting evidence of a causal relationship. Neither the MR-Egger intercept nor the MR-PRESSO global test detected evidence of potential horizontal pleiotropy (p > 0.05). In sensitivity analyses, Cochran's Q test indicated no evidence of heterogeneity (p > 0.05) among outlier-corrected IVs (See Methods), and leave-one-out analyses identified no single genetic variant that influenced the causal estimate. Full results for the MP-SA MR analysis are provided in Supplementary Table 13.

For the effect of MP on SD, an OR of 1.11 [95% CI = 1.03–1.21] and p = 0.011 were obtained by IVW analysis (Fig. 6b). There was no evidence of horizontal pleiotropy, heterogeneity, or influential outlier IVs based on the MR-Egger intercept, MR-PRESSO global test, Cochran's Q statistic, or leave-one-out analyses (Supplementary Table 13). Although the WM and MR-Egger methods did not reach statistical significance, their estimates were directionally consistent with the IVW result (WM OR = 1.07, p = 0.23; MR-Egger OR = 1.24, p = 0.29).

Reverse MR analyses were conducted to evaluate the potential causal effects of SA and SD on MP. Due to the limited number of GWAS SNPs available for both SA and death, a conservative set of instrumental variables was defined using a relaxed significance threshold (p < 5e-5). Although we observed evidence supporting a putative causal effect of SA liability on MP, the estimated effect size was modest (OR = 1.01, CI = 1.01–1.02, p = 8.46e-07) based on outlier-corrected IVs. In contrast, no significant causal association was observed for SD on MP (Supplementary Table 13).

### Mediation analyses in multidimensional pain and suicide attempt

To identify potentially mediating phenotypes in the relationship between MP and SA, we conducted two-step MR analyses across 23 health-related phenotypes spanning five conceptual domains. These phenotypes were defined *a priori* based on reported clinical associations with both MP and suicidal behavior. This analysis was limited to SA because the MR result for MP and SD was underpowered.

Among these phenotypes, 14 were identified as potential mediators (Fig. 6c and Supplementary Table 14). Post-traumatic stress disorder (PTSD) accounted for the largest estimated proportion of the total effect of MP on SA (47.67%), followed by major depressive disorder (MDD; 29.60%). Notably, all substance-related behaviors-including cannabis use disorder (CUD; 13.53%), opioid medication (N02A; 13.23%), alcohol use disorder (AUD; 8.30%), smoking behavior (7.21%), and problematic alcohol use (PAU; 6.33%)-were identified as potential mediators. Within the psychological well-being domain, there was evidence supporting mental well-being spectrum, feeling miserable, feeling tense, feeling lonely, and irritable mood as mediators, with estimated mediation proportions ranging from 3.91% to 12.59%. In the psychiatric disorder domain, bipolar disorder (BIP; 8.10%) were additionally identified as mediators, with no mediating evidence observed for attention-deficit/hyperactivity disorder (ADHD) or schizophrenia (SCZ). Among the Big Five personality traits, only neuroticism showed evidence of being a mediator (15.92%).

## Discussion

This study delineates the shared genetic architecture underlying MP and suicidal behaviors-suicide attempt and suicide death-through comprehensive cross-trait genetic analyses. A substantial proportion of trait-influencing variants inferred by the MiXeR model underlying SA and SD were shared with variants influencing MP. Consistent with this observed polygenic overlap, we identified 76 and 10 independent genomic loci in MP jointly associated with SA and SD, respectively. Notably, the majority of the loci for both SA (51 loci) and SD (8 loci) did not reach genome-wide significance in the original single-trait GWAS. Integration of multi-omics resources further provided functional insight into molecular pathways potentially underlying shared susceptibility to pain and suicidal behaviors. Furthermore, MR analyses extended these associations to genetically informed causal relationships between MP and SA, identifying potential mediation roles through addiction and psychiatric phenotypes. These findings advance our understanding of a mechanistic intersection linking MP liability and suicidal behaviors via shared architecture, pleiotropic loci, and putative causal pathways.

Despite the modest model fit for SD in the global MiXeR analysis, the SNP-heritability estimate remained statistically robust (h^2^ = 0.129, SE = 0.012, Z-score = 10.75). This significant polygenic signal supports the inclusion of SD in exploratory bivariate analyses as a severe-endpoint extension, demonstrating a pattern broadly consistent with SA. The comparatively modest model support is consistent with reduced statistical power for this rarer phenotype. However, locus-level analyses using conjFDR provided complementary evidence of shared genetic liability between MP and SD.

Our analyses utilized a GWAS meta-analysis of MP that focused on the shared and unique genetic contributions of the intensity, distribution, and diagnosis of pain and provide a more comprehensive view of the genetic underpinnings of multidimensional pain^24^. Previous epidemiological and cross-sectional studies have consistently reported associations of multifaceted pain and suicidal behaviors^3,4,6-10,36-42^. Population-based studies have shown elevated suicide risk among individuals with chronic pain conditions, even after controlling for psychiatric comorbidities^4^. Importantly, pain characteristics, such as severity, intensity, duration, and frequency, have also been linked to suicidality, potentially accounting for the heterogeneity and complexity observed in associations between specific chronic pain diagnoses and suicide risk^37-39,41^, although some studies have reported weak or inconsistent associations between certain pain characteristics^3^. Such inconsistencies may indicate the presence of indirect, mediational pathways through which chronic pain conditions influence suicide risk, and suggest that broader, multidimensional aspects of pain may provide a better understanding of suicide-related mechanisms than individual pain characteristics or diagnoses alone.

The polygenic overlap analysis revealed mixed effect directions among variants shared between MP and SA, suggesting etiologic heterogeneity underlying their relationship. In contrast, for SD, the majority of shared variants showed concordant effect directions, accompanied by positive local genetic correlations in the LAVA analysis. These contrasting patterns may reflect differences in underlying etiologic mechanisms between the two suicidal behaviors. SA likely represents a broader liability encompassing multiple pathways, including those related to psychiatric conditions, whereas SD may be driven by more specific biological pathways associated with severity and lethality. Nevertheless, the apparent predominance of concordant effects for SD warrants cautious interpretation and should be evaluated in future larger-scale GWAS.

In addition, the Q-Q plot showed conditional enrichment in both directions between MP and SA, suggesting the presence of a bidirectional genetic overlap (not necessarily implying bidirectional causal relationships). This finding is consistent with that of a previous study in which there was a bidirectional association between persistent or recurrent pain trajectory and suicidality^8^. A potential biological mechanism underlying this shared genetic direction involves altered physiological sensitivity in neural stress-regulatory systems.

Gene-set enrichment analysis of genes mapped to shared genetic loci between MP and SA identified glutamatergic and GABAergic synapse-related GO terms, consistent with prior findings from original GWAS for MP and pain intensity that reported involvement of the GABAergic system^24,43^. These synapse-related gene sets reflect excitatory and inhibitory neurotransmitter systems that play a central role in both chronic pain and suicidal behaviors by maintaining the balance of neural excitability within pain- and emotion-related circuits^13,44-46^. The GABAergic system has been suggested to contribute to the comorbidity between chronic pain and depression, a clinical context strongly associated with suicidal behavior^47^. Notably, the hypothalamic-pituitary-adrenal (HPA) axis is a downstream stress-regulatory system that is partly modulated by GABAergic signaling and has been proposed to be a mediating pathway linking pain with psychological distress and psychiatric disorders associated with suicide risk^47,48^. Chronic pain can act as a sustained biological stressor, leading to dysregulation of the HPA axis, while HPA axis dysfunction has been associated with altered pain sensitivity and reduced pain thresholds^49-53^. Thus, these observations support the presence of a stress-pain feedback loop that may contribute to the observed bidirectional genetic overlap between MP and suicidal behaviors.

The SMR analysis was aligned with these observations, prioritizing 10 genes as putative effector genes, the majority of which are implicated in brain and nervous system functions. Among these, *DRD2* provides biological context relevant to the behavioral framework observed in the SMR analyses. *DRD2* encodes the dopamine receptor D2 and maps to the most significant shared locus (rs2514218) identified by conjFDR, representing a central component of the dopaminergic system. *DRD2* plays a critical role in reward processing by modulating neuronal activity through dopamine signaling, and genetic variants linked to this gene have been extensively associated with psychiatric and substance-related behaviors, as well as pain-related traits^54-57^. Although this shared locus (rs2514218) has been detected in previous single-trait GWAS for both MP and SA, our integrative cross-trait analyses extend these findings by demonstrating that *DRD2* may reflect a shared genetic mechanism underlying both traits. In addition, *ANNK1* and *TTC12*, which were also prioritized in the SMR analyses, are part of a gene cluster (*ANNK1-TTC12-DRD2*) on chromosome 11q. This cluster has been associated with addiction-related traits, including alcohol dependence^58^, heroin dependence^59^, and smoking behavior^60^, as well as neurodevelopmental phenotypes, such as ADHD^61^. Collectively, pathways involving these genes highlight dopaminergic and addiction-related neurobiological mechanisms that may plausibly contribute to the behavioral mediation observed between MP and SA.

Beyond stress-regulatory and dopaminergic mechanisms, convergent evidence from enrichment analyses and SMR gene prioritization further implicated broader neurodevelopmental processes. Gene-set enrichment analyses identified pathways related to neural precursor cell proliferation, synaptic-related pathways, signaling by MST1, axon guidance, and semaphorin-related pathways, suggesting additional shared biological relevance to MP and suicidal behaviors^62-67^. In addition, other prioritized genes, including *PTBP2*^68,69^, *KIF1A* ^70,71^, *SEMA6D*^72^, and *FURIN*^73,74^ are involved in neuronal and synaptic functions and have been linked to neuropsychiatric traits, further supporting cross-trait biological convergence. Together, these convergent findings may suggest shared neurodevelopmental processes linking pain liability with suicide risk.

Notably, one gene, *FOXP2*, was consistently identified across both the MP-SA and MP-SD analyses, suggesting shared genetic relevance across suicidal phenotypes. *FOXP2* encodes a transcription factor that plays an important role in brain development, including regulation of synapse formation, being implicated in chronic pain phenotypes and suicide risk^75-77^. *FOXP2* has also been repeatedly associated with substance use disorders, particularly cannabis use disorder, across multiple genome-wide association studies^54,78,79^, as well as with neurodevelopmental conditions^80^. These broader associations suggest that *FOXP2* may reflect more general neurobiological vulnerability rather than being specific to chronic pain and suicide.

This study applied bidirectional MR to explore potential genetic causality between MP and suicidal behaviors. Our findings provide evidence consistent with a potential genetically causal relationship between MP and both SA and SD, extending prior work^17^ by demonstrating that there is an association between multidimensional pain and suicidal behaviors. It suggests the relationship is not limited to a single pain condition. Moreover, we observed evidence suggestive of a bidirectional causal genetic association between MP and SA. However, the reverse MR analysis showed modest effect sizes. These findings suggest bidirectional effects with asymmetric strength, although the reverse direction should be interpreted cautiously given the limited statistical power and the use of a relaxed instrument selection threshold (p < 5e-5), which may have introduced pleiotropic effects from genetically correlated traits. Future analyses, using even larger GWAS discovery meta-analyses, are expected to enable more rigorous evaluations of potential bidirectional causal relationships.

In epidemiological studies, chronic pain has been associated with a range of health-related phenotypes that are themselves associated with risk for suicidal behaviors, including psychiatric conditions^40^. For example, chronic pain is associated with depressive symptoms, and major depressive disorder has been examined as a potential mediator linking multisite chronic pain to suicidal behaviors^17^. In addition to psychiatric conditions, behavioral phenotypes, such as substance-related behaviors, and personality traits are observed to be more prevalent among individuals with chronic pain who die by suicide, indicating their potential importance in shaping the pain-suicide relationship^2^. Understanding the contribution of these phenotypes is particularly relevant for identifying modifiable risk factors that may reduce suicide risk through behavioral interventions and other non-pharmacological strategies among pain-affected populations. Consistent with this framework, the original GWAS of MP reported putative causal relationships between pain and multiple health-related risk traits, including MDD, neuroticism, PTSD, insomnia, and opioid use. These observations provided the rationale for a systematic investigation of the mediating roles of health-related risk traits in the association between MP and suicidal behaviors. Thus, we examined the potential mediating roles of 23 health-related phenotypes across five conceptual domains, selected *a priori* based on reported clinical and epidemiological associations as general risk factors for suicidal behaviors in the context of MP. Psychiatric traits, such as PTSD and MDD, emerged as strong mediators, which is in line with prior studies identifying MDD as a key intermediate linking chronic pain and suicidality^17,36^. The strong mediating roles may reflect the dynamic interplay between physical and psychological dimensions of pain, in line with concepts such as somatization and affective pain processing.

Notably, all examined substance-related behaviors, including CUD, opioid medication use, AUD, smoking, and PAU, showed evidence of potential mediation of the causal genetic relationship between MP and SA, consistent with the involvement of stress- and reward-related neurobiological pathways described above. These findings suggest that substance-related behaviors, representing a major subset of harmful health-related habits, may be relevant behavioral targets for suicide prevention among individuals with genetic liability to chronic pain. Among psychiatric disorders, BIP additionally demonstrated significant mediating roles. In contrast, ADHD and SCZ were not supported as mediators, likely due to absence of genetically causal effects from MP on these disorders. Furthermore, in an observational study, mental defeat heightened future suicide risk in individuals with chronic pain^36^. In line with this, our analyses identified evidence of causal mediation through several mental well-being-related phenotypes, including mental wellbeing spectrum measures, feeling miserable, feeling tense, feeling lonely, and irritable mood.

In addition, neuroticism was the only Big Five personality trait that mediated the causal relationship between MP and suicidal attempt. Taken together, these findings highlight a broad set of mediating pathways. By delineating these mediators, our study advances understanding of potential causal mechanisms underlying this relationship and underscores opportunities for both pharmacological and non-pharmacological strategies to mitigate suicide risk in pain-affected populations. Although we did not perform formal drug target enrichment analyses, the shared biological mechanisms identified here lay the groundwork for future therapeutic target discovery.

We acknowledge several general limitations of our study. First, our study relied on GWAS summary statistics generated from individuals of European-like genetic ancestry (EUR). This limited ancestral diversity may restrict the generalizability of our findings to other populations. Second, the GWAS for SD was conducted in a relatively small sample size, resulting in limited statistical power. Consequently, the genetic architecture shared between MP and SD could not be fully characterized. For example, the MiXeR bivariate model between these two traits did not provide a sufficient model fit, as indicated by negative AIC values, preventing reliable differentiation between the minimum and maximum possible degrees of genetic overlap. Therefore, the overlap estimates involving SD should be interpreted with caution. In addition, functional gene enrichment and mediation analyses for SD were not conducted due to insufficient statistical power. Future GWAS with larger sample sizes for SD will be necessary to replicate and validate the observed associations with MP. Third, our study could not quantify the contribution of additional factors, such as psychiatric or medical comorbidities, to the identified genetic overlap estimated from MiXeR and conjFDR analyses. This limitation arises because the original GWAS summary statistics did not account for heterogeneity related to comorbidities, and because the analyses were restricted to a bivariate framework. Fourth, the validity of MR depends on key assumptions, including the absence of horizontal pleiotropy and unmeasured confounding. Although we applied multiple sensitivity analyses and stringent filtering criteria to mitigate potential violations of these assumptions, they cannot be entirely excluded, particularly, given that the biological functions of many associated SNPs remain incompletely characterized. For the meditation analyses, residual confounding by additional unmeasured variables remains possible. Fifth, suicide ideation was not examined in this study, although future studies could leverage the new GWAS for this trait^81^. Given that suicide ideation is more prevalent than SA in individuals with chronic pain^37^, its potential role as a mediator of the MP-SA relationship could be explored in future studies. Finally, given known sex differences in the prevalence of pain, SA, and SD, future studies are needed to examine potential sex-specific architectures and mediation patterns underlying these associations.

In conclusion, our integrative cross-trait genetic analyses demonstrate a shared genetic architecture between MP and suicidal behaviors, characterized by extensive polygenic overlap and shared genetic loci. By integrating multiple genetic approaches, our findings provide convergent evidence for interconnected biological and behavioral pathways linking pain liability and suicidal behaviors. These findings extend prior models of the pain-suicide relationship by integrating genetic liability, behavioral mediation, and neurobiological context, and underscore substance-related behaviors as modifiable behavioral pathways within this framework. This work supports a multilevel liability model in which shared polygenic influences on stress-regulatory, synaptic, and reward-related systems confer vulnerability to multidimensional pain, which may, through psychiatric and behavioral intermediaries, increase risk for suicidal behaviors. Together, our findings provide insights that may inform future work on the identification of both potential non-pharmacological and pharmacological intervention strategies, as well as approaches to suicide risk stratification among individuals with a genetic predisposition to pain.

## Methods

### GWAS summary data sets

The GWAS summary statistics for multidimensional pain were generated from a recent meta-analysis involving the largest of a pain phenotype to date (*N* = 1,235,695 individuals of European-like ancestry (EUR))^24^. This GWAS of MP captures a broad, general genetic liability to chronic pain as a latent construct through meta-analysis integrating signals from multiple pain dimensions, including pain intensity, chronicity, spatial distribution, and diagnosis-based pain codes, across diverse pain assessment frameworks.

Additionally, we analyzed summary statistics from a GWAS meta-analysis of SA including 787,974 (cases = 30,517 and controls = 757,457) EUR individuals^25^. The original GWAS meta-analyzed 15 cohorts. However, because two cohorts (University of Utah and Columbia University) included suicide death samples, we used summary statistics from the meta-analysis excluding those cohorts. The SD GWAS was conducted in 18,223 (cases = 3,413 and controls = 14,810) EUR individuals^26^, and these samples were independent from those included in the SA GWAS. All GWAS summary statistics were preprocessed and harmonized using the “*sumstats.py*” script (https://github.com/precimed/python_convert/). Genetic loci within the MHC region (chr6:26000000–34000000) were excluded to avoid potential bias arising from the region’s complex LD structure.

### Polygenic overlap analysis

To characterize shared genetic architecture at both genome-wide and local levels, we first estimated genome-wide genetic correlations (*r_g_*) using LD score (LDSC) regression. The MiXeR (v1.3) framework^28^ was employed to quantify the polygenic overlap between traits, by modeling a mixture of concordant and discordant genetic effects that may not be fully reflected by LDSC estimates. Univariate MiXeR analyses were conducted to estimate SNP-based heritability and polygenicity, defined by the number of trait-influencing "causal" variants, for each phenotype. Heritability was calculated by aggregating the effects of these variants, and polygenicity was defined as the estimated number of such variants. Model fit was assessed using the Akaike information criterion (AIC) values by comparing the full mixture model fit with an infinitesimal model assuming that all variants have small effect sizes^32^. A positive AIC difference indicates sufficient power of GWAS summary statistics to yield reliable estimates with adequate model performance.

Bivariate MiXeR analyses were subsequently performed to estimate trait-specific and shared variants by extending the univariate models to each trait pair, including MP-SA and MP-SD. Bivariate model fit was evaluated with two AIC differences: AIC_min_ by comparing the best-fitting model with a model assuming the minimum possible polygenic overlap, and AIC_max_ comparing the best-fitting model with a model assuming the maximum possible overlap^32^. Positive values of both AIC_min_ and AIC_max_ indicate that the fitted bivariate model is distinguishable from both extreme overlap scenarios, supporting adequate model fit for estimating shared polygenic architecture. A dice coefficient defined as the proportion of shared variants of the total number of trait-influencing variants was calculated, and the number of variants for both trait-specific and shared were displayed in the Venn diagram by MiXeR.

MiXeR additionally generated conditional quantile-quantile (Q-Q) plots to visualize cross-trait genetic enrichment. In these plots, nominal p-values for one trait were stratified by progressively more stringent significance thresholds of the secondary trait (p < 1, p < 0.1, p < 0.01, and p < 0.001). A progressive leftward deflection from the empirical null line indicates the presence of pleiotropic enrichment between the two traits. LD structure for the MiXeR analysis was derived from the European ancestry samples of the 1000 Genomes EUR reference panel.

Local genetic correlation analyses were performed using LAVA (v0.1.5), based on 2,500 independent LD blocks defined by the LAVA partitioning algorithm developed by the LAVA team (https://github.com/cadeleeuw/lava-partitioning). The aim of this analysis was to assess the distribution and directional heterogeneity of local genetic correlations across the genome, as suggested by the mixed-effect overlap observed in MiXeR analyses, rather than to identify specific genomic hotspots. Accordingly, bivariate local genetic correlations were estimated across LD-based regions that showed nominally significant univariate local SNP-heritability (p < 0.05). A total of 1,948 and 1,329 genomic regions were included in the analyses for the MP-SA and MP-SD pairs, respectively, as returned by the LAVA framework. P-values of local genetic correlations across LD-based genomic regions were adjusted for multiple testing using FDR. Significant LAVA regions were defined at FDR < 0.05, which FDR < 0.1 considered suggestive.

### Identification of specific shared genetic loci

We conducted conjFDR (https://github.com/precimed/pleiofdr) analyses to identify specific genetic loci jointly associated with MP and SA, as well as with MP and SD. LD structure was estimated using the 1000 Genomes Project EUR reference panel, and loci with conjFDR < 0.05 were considered significant.

Given the conjFDR results, we used the Functional Mapping and Annotation (FUMA) platform^34^ to define lead SNPs and to perform functional annotation using the 1000 Genomes Project EUR reference panel. Within each LD block (r^2^ > 0.2), the SNP with the minimum conjFDR value was defined as the lead SNP, and the lead SNPs located within 250 kb of each other were merged into a single genomic risk locus in FUMA.

All candidate SNPs were annotated using three functional resources: 1) 15-core chromatin state annotations^82^, 2) RegulomeDB scores^83^, and 3) CADD scores^84^. Chromatin states ≤ 7 indicate open chromatin regions with potential regulatory activity, RegulomeDB scores ≤ 2 provide evidence of regulatory function, including transcriptional factor binding, and CADD scores > 12.37 suggest putatively deleterious variants.

“Novel” loci refer to loci that are novel in shared cross-trait associations between MP and SA or SD, rather than aiming to define loci that are entirely newly discovered for each individual trait. We defined a locus as novel when no SNP within the LD block (defined around the lead SNP with r^2^ = 0.2) reached genome-wide significance (P < 5e-8) in the original GWAS for either MP or SA/SD. For SA, we used summary statistics from the GWAS excluding the Utah and Columbia cohorts due to the inclusion of SD cases in those cohorts. To avoid overestimating novelty, genome-wide significance was evaluated in both the full-cohort GWAS and the cohort-excluded subset GWAS when determining novelty.

### Gene mapping and functional annotation

Using FUMA, candidate SNPs were mapped to protein-coding genes according to three complementary approaches: 1) positional mapping within a 10 kb window, 2) functional annotation with ANNOVAR^33^, and 3) eQTL mapping based on cis-eQTLs data from GTEx project (v8)^85^. In addition, sQTL mapping was performed using cis-sQTL data from the GTEx project.

Functional over-representation analyses were conducted to evaluate the biological implications of the mapped genes using ConsensusPathDB (CPDB)^35^, integrating multiple pathways (e.g., KEGG and Reactome) and gene ontology (GO) databases. In addition, enrichment of GWAS traits among the mapped genes was evaluated using the GENE2FUNC module in FUMA. Gene sets with FDR adjusted q-values < 0.05 were considered statistically significant.

### Gene prioritization

To prioritize putatively functional genes among genes mapped to shared loci described above, we applied summary data-based Mendelian randomization (SMR) approach^30^, integrating multi-omics quantitative trait locus (QTL) data with GWAS summary statistics for each of MP, SA, and SD using SMR-Portal^86^. Four categories of QTLs were examined in the SMR analyses: eQTLs, sQTLs, mQTLs, and pQTLs to assess gene expression, alternative splicing levels, DNA methylation, and plasma protein levels, respectively. The QTL data from tissues relevant to pain and suicidal behaviors, including brain, blood, skeletal muscle, and tibial nerve, were analyzed. The eQTL data were obtained from BrainMeta^87^ for brain tissue (n = 2,865), from eQTLGen^88^ for blood (n = 31,684), and from the GTEx project for tibial nerve (n = 619) and skeletal muscle (n = 803). The sQTL data were obtained from BrainMeta for brain tissue (n = 2,865) and from the GTEx project for whole blood (n = 755), tibial nerve (n = 619), and skeletal muscle(n = 803). BrainMeta brain eQTL data were selected due to their larger sample size, while GTEx brain eQTLs were not included to avoid statistical redundancy. GTEx whole blood sQTL data were used in place of eQTLGen blood data that was used for eQTLs, as there is no sQTL information in the eQTLGen data. The mQTL data were obtained from BrainMeta for brain tissue (n = 1,160) and from McRae for blood (n = 1,980). The pQTL data were obtained from INTERVAL^89^ (n = 3,301) and the Fenland^90^ (n = 10,708), both for blood. SMR associations were considered significant when they passed FDR < 0.1 in both MP and SA within the same omics layer. To claim pleiotropic associations from those driven by LD, subsequent heterogeneity in dependent instruments (HEIDI) analysis was applied, and associations with *P*_HEIDI_<0.01 were excluded.

### Mendelian randomization analysis to infer causal relationship

We conducted bidirectional, two-sample MR analyses to investigate potential causal relationships between MP and SA or SD using the MendelianRandomization R package^91^. Genetic instrumental variables (IVs) were defined through a series of rigorous quality control steps following standard MR assumptions^92^.

First, genome-wide significant SNPs (p < 5e-8) were extracted from GWAS summary statistics of the exposure. LD clumping was then performed to obtain independent SNPs using a threshold of r^2^ = 0.001 and 1,000 kb window based on the 1000 Genomes Project EUR reference panel. F-statistics were calculated for each IV using the formula^93^ (β/SE)^2^, and SNPs with F-statistics < 10, which may introduce weak instrument bias, were excluded. To further minimize confounding, we excluded SNPs associated with potential confounders plausibly relevant to both MP and suicidal behaviors, as defined using the GWAS Catalog^94^, including smoking, sleep duration, sleep traits, insomnia complaints, alcohol consumption, HDL cholesterol levels, LDL cholesterol levels, diastolic blood pressure, systolic blood pressure, type 2 diabetes, chronic kidney disease, body mass index, educational attainment, self-reported tiredness, and household income. SNPs were considered overlapping if any SNP withing the LD block (r2 = 0.2) was reported for these traits in the GWAS Catalog. Steiger directionality tests^95^ were applied to ensure that each IV showed a stronger association with the exposure than with the outcome, thereby retaining IVs primarily associated with the exposure. In addition, the MR pleiotropy residual sum and outlier (MR-PRESSO)^96^ global test was used to detect and remove outlier IVs, yielding the final set of instruments for MR analyses. Summary statistics for IVs were harmonized using the “harmonise_data” function in the TwoSampleMR R package^97^.

The inverse variance weighted (IVW) method was used as the primary MR approach to estimate causal effects. Two complementary MR methods-the weighted median (WM) and MR-Egger^98^-were additionally applied to assess the robustness of the results. Sensitivity analyses were conducted to evaluate potential violations of MR assumptions, including assessment of directional horizontal pleiotropy using the MR-Egger intercept (p < 0.05) and MR-PRESSO, and heterogeneity among IVs using Cochran's Q statistic. Leave-one-out analyses were also performed to examine the influence of a single IV on the overall causal estimates. When outliers or substantial heterogeneity were detected, MR analyses were performed using a corrected set of IVs after excluding the identified outlier instruments.

### Mediation analysis

Two-step MR mediation analyses were subsequently performed to identify potential mediators linking MP to suicidal behaviors. Candidate mediators were systematically examined across five conceptual domains: 1) psychological well-being traits, 2) psychiatric disorders, 3) substance-related behaviors, 4) personality traits, and 5) sleep-related trait, encompassing 23 health-related phenotypes. These phenotypes were defined based on *a priori* knowledge of their associations with both MP and suicidal behaviors. Detailed information on the included phenotypes is provided in Supplementary Table 15.

For each potential mediator, we first conducted MR analyses to estimate the association between MP (exposure) and the mediator. We then performed MR analyses to estimate the association between the mediator (as exposure; e.g., alcohol use disorder) and the suicidal outcome (outcome), following the same MR workflow described above. The indirect effect was estimated as the product of the MR effect estimates for the exposure-mediator and mediator-outcome associations. The direct effect of MP on suicidal behaviors was calculated as the difference between the total MR effect and the estimated indirect effect.

Statistical significance of the indirect effect was assessed using a bootstrapping approach (10,000 resamples) to estimate 95% confidence intervals and p-value, and the ratio of the indirect effect to the total effect was used to quantify the proportion mediated^99^.

## Supplementary Material

This is a list of supplementary files associated with this preprint. Click to download.

- SuppleTables.xlsx
- SuppleFigures.pdf

## Data Availability

The summary statistics for multidimensional pain, suicide attempt, and suicide death analyzed in this study were generated from previous studies^24-26^. Summary statistics of the 23 health-related phenotypes for the mediation analyses are publicly available, with details provided in Supplementary Table 15.

## Code Availability

LAVA: https://github.com/josefin-werme/LAVA; MiXeR: https://github.com/precimed/mixer; LDSC: https://github.com/bulik/Idsc; pleioFDR for conjFDR analysis: https://github.com/precimed/pleiofdr; FUMA: https://fuma.ctglab.nl/; ConsensusPathDB: http://cpdb.molgen.mpg.de/MCPDB; SMR portal: https://yanglab.westlake.edu.cn/smr-portal; MendelianRandomization R package: https://cran.r-project.org/web/packages/MendelianRandomization/index.html.
